# The LIN28B/TGF-β/TGFBI feedback loop promotes cell migration and tumour initiation potential in cholangiocarcinoma

**DOI:** 10.1038/s41417-021-00387-5

**Published:** 2021-09-21

**Authors:** Nattapong Puthdee, Sira Sriswasdi, Trairak Pisitkun, Sutheera Ratanasirintrawoot, Nipan Israsena, Pisit Tangkijvanich

**Affiliations:** 1 Department of Biochemistry, Faculty of Medicine, Chulalongkorn University, Bangkok, Thailand; 2 Department of Research Affairs, Faculty of Medicine, Chulalongkorn University, Bangkok, Thailand; 3 Center of Excellence for Stem Cell and Cell Therapy, Faculty of Medicine, Chulalongkorn University, Bangkok, Thailand; 4 Department of Pharmacology, Faculty of Medicine, Chulalongkorn University, Bangkok, Thailand; 5 Center of Excellence in Hepatitis and Liver Cancer, Faculty of Medicine, Chulalongkorn University, Bangkok, Thailand

**Keywords:** Cell biology, Biomarkers

## Abstract

Cholangiocarcinoma (CCA), a lethal malignancy of the biliary epithelium, is the second most common primary liver cancer. The poor prognosis of CCA is due to the high rate of tumour invasion and distant metastasis. We found that the RNA-binding protein LIN28B, a known regulator of microRNA biogenesis, stem cell maintenance, and oncogenesis, is expressed in a subpopulation of CCA patients. To further investigate the potential role of LIN28B in CCA pathogenesis, we studied the effect of LIN28B overexpression in the cholangiocyte cell line MMNK-1 and cholangiocarcinoma cell lines HuCCT-1 and KKU-214. Here, we show that enhanced LIN28B expression promoted cancer stem cell-like properties in CCA, including enhanced cell migration, epithelial-to-mesenchymal transition (EMT), increased cell proliferation and spheroid formation. Proteomic analysis revealed TGF-β-induced protein (TGFBI) as a novel LIN28B target gene, and further analysis showed upregulation of other components of the TGF-β signalling pathway, including TGF-β receptor type I (TGFBRI) expression and cytokine TGFB-I, II and III secretion. Importantly, the small molecule TGF-β inhibitor SB431542 negated the effects of LIN28B on both cell migration and clonogenic potential. Overexpression of TGFBI alone promoted cholangiocarcinoma cell migration and EMT changes, but not spheroid formation, suggesting that TGFBI partially contributes to LIN28B-mediated aggressive cell behaviour. These observations are consistent with a model in which TGF-β and LIN28B work together to form a positive feedback loop during cholangiocarcinoma metastasis and provide a therapeutic intervention opportunity.

## Introduction

Cholangiocarcinoma (CCA) is a biliary tract cancer with one of the highest mortality rates among all cancers and has an increasing incidence rate worldwide [1]. The incidence of CCA is differentially distributed in differing geographical areas, especially in Northeast Thailand, which has the highest incidence and is closely related to liver fluke-induced CCA [2, 3]. CCA is recognised as a devastating cancer with limited therapeutic options and poor prognosis. Molecular profiling of CCA revealed a high degree of intertumour and intratumour heterogeneity [4]. Based on an immunostaining study, it has been suggested that CCA may contain cancer stem cell-like subpopulation switches that have high tumour-initiating potential and chemoresistance and that could be responsible for relapse after treatment [5]. Understanding the mechanisms governing CCA aggressiveness and drug resistance could lead to new therapeutic targets for the treatment of this cancer.

LIN28 is an RNA-binding protein that plays pivotal roles in development, stem cell maintenance, energy metabolism and tumorigenesis [6, 7]. LIN28A and its paralogue, LIN28B, have been known to block let-7 miRNA maturation and modulate mRNA translational efficacy by binding to mRNA targets [8–11]. The expression of LIN28 is generally found in developing foetal tissues and then disappears after birth. It has been reported that the LIN28A and LIN28B genes are reactivated in many human malignancies, such as breast cancer, colon cancer, lung cancer, ovarian cancer and hepatocellular carcinoma (HCC), and their expression is correlated with poor clinical disease outcomes [12]. LIN28 overexpression was reported to be sufficient to initiate HCC in transgenic mouse models [13]. Mechanisms proposed to be responsible for oncogenic transformation of LIN28 include derepression of let-7 targets such as cMyc, RAS and HMGA2 oncogenes [14–16] and let-7-independent translational control of genes that regulate the cell cycle and stemness, such as cyclin A, CDK4, and OCT4 [16]. In cholangiocarcinoma, downregulation of a member of the let-7 miRNA family in human CCA tissues compared to normal bile duct tissues has been reported [17, 18]. In a mouse model of cholestasis-associated CCA, LIN28B expression was increased, suggesting that the LIN28 pathway may play a role in the pathogenesis of CCA [19]. Nonetheless, the role of the LIN28 pathway in CCA has not been studied.

In this study, we report LIN28B expression in human CCA samples. We investigated the roles of LIN28B in regulating cell proliferation, stemness and cell migration by overexpressing LIN28B in cholangiocyte and CCA cell lines. Our work demonstrates that LIN28B promotes many cancer stem cell-like properties in CCA. We show that some of the LIN28B effects are mediated through enhanced TGF-β signalling and TGFBI secretion. Our results suggest that feedback collaboration between LIN28B and TGF-β plays a role in cholangiocarcinoma metastasis and provide a novel target for therapeutic intervention for CCA.

## Results

### LIN28B is overexpressed in a subset of cholangiocarcinoma patients

**Fig. 1:**
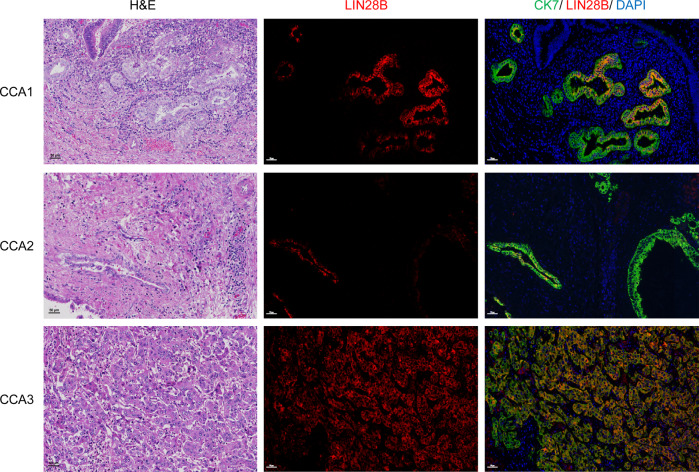
LIN28B is overexpressed in a subpopulation of human CCA tissues. Representative pictures of LIN28B immunofluorescence (IF) and H&E staining in three human CCA patient tissues. In IF staining, green indicates bile duct marker CK7-stained cells, red indicates LIN28B-stained cells and blue indicates nuclear staining.

LIN28A and LIN28B are expressed in the liver throughout development but disappear after birth [20]. Aberrant expression of LIN28B has been reported in HCC [21]. To investigate whether the LIN28 pathway is dysregulated in CCA, we first retrospectively examined LIN28 expression in paraffin-embedded surgical tissues from 20 patients pathologically diagnosed with CCA at our institute. While we could not detect LIN28A-stained cells in either tumour or normal tissue, heterogeneous LIN28B protein expression in a subpopulation of cancer cells was found in 3 CCA patient tissues. Immunofluorescence staining demonstrated that LIN28B was predominantly localised in the cytoplasm of bile duct epithelial marker (CK7)-positive cells (Fig. 1). Since ectopic expression of LIN28B in other cancers usually correlates with cancer stem cell-like properties and poor prognosis [12], our data provide supporting evidence for investigating the potential roles of LIN28B in the CCA context.

### Overexpression of LIN28B promotes MMNK-1 proliferation and increases tumour-initiation capacity

**Fig. 2:**
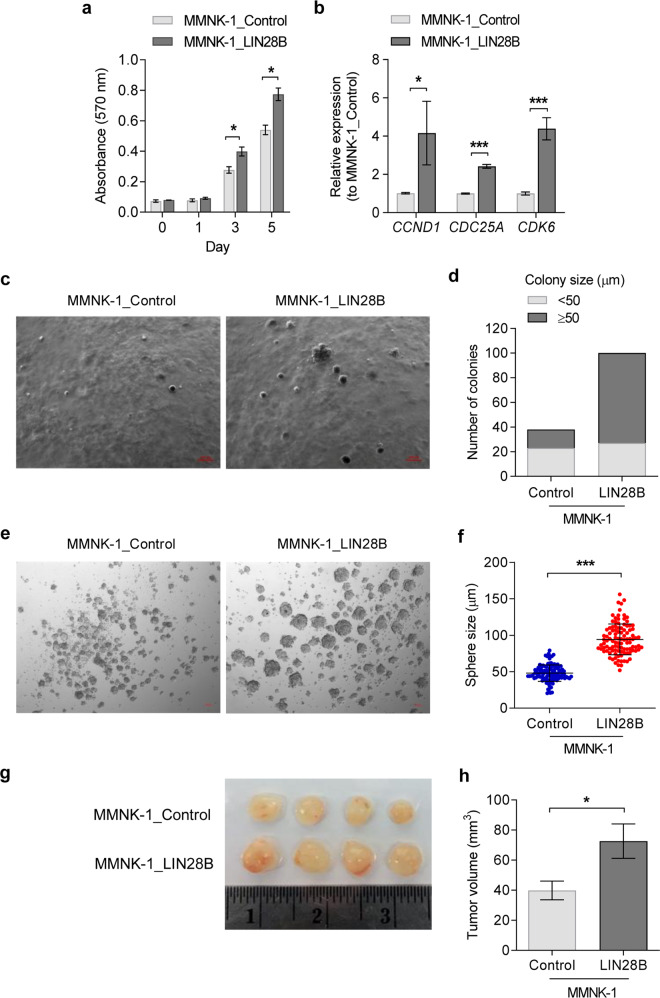
Overexpression of LIN28B enhances cell proliferation and induces tumour initiation capacity. MTT assays were used to measure cell viability on days 0, 1, 3 and 5 in the control and LIN28B-overexpressing MMNK-1 cell lines (**a**). The gene expression levels of the CCND1, CDC25A and CDK6 genes were assessed by RT-qPCR (**b**). Representative images of the soft agar colony formation assay of LIN28B-overexpressing MMNK-1 cells compared to the control (**c**). Total colonies from nine focal planes of the acquired images were counted, and their size was measured (**d**). The sphere-forming capability of LIN28B-overexpressing MMNK-1 cells is shown in **e**, spheroids were counted, and the size was measured (**f**). Representative tumours from control and LIN28B-overexpressing MMNK-1 xenograft mice (*n* = 4 in each group) (**g**) and tumour volume at day 28 of the experiment (**h**). Data are presented as the mean ± SD, *n* = 3 and mean ± SEM for **h**. Statistical significance is indicated by *P < 0.05, **P < 0.01, ***P < 0.001.

A previous study demonstrated that LIN28B overexpression in hepatocytes was sufficient to induce HCC [13]. To elucidate the roles of LIN28 in the regulation of cholangiocyte properties, we constitutively overexpressed human LIN28B in immortalised cholangiocyte cells, MMNK-1. We confirmed that MMNK-1_LIN28B cells expressed high levels of LIN28B and reduced levels of the let-7 family by western blot and RT-qPCR (Supplementary Fig. 1). First, we observed that LIN28B significantly increased the proliferation rate of MMNK-1 cells (Fig. 2a). We then examined known LIN28/let-7 target genes that regulate the cell cycle and proliferation, *CCND1*, *CDC25A* and *CDK6*. As expected, the expression of these genes was significantly increased in MMNK-1_LIN28B cells compared to control cells (Fig. 2b). The effect on cholangiocyte transformation of LIN28B was addressed using a soft agar colony forming assay, which measures the cell capability for anchorage-independent growth. We found that LIN28B significantly increased both colony numbers and size compared with control MMNK-1 cells (Fig. 2c, d). Moreover, we determined the capability of LIN28B to induce sphere formation in MMNK-1 cells and found that the spheroid size in LIN28B-overexpressing cells was significantly larger than that in control cells (Fig. 2e, f). Upon transplantation into NSG mice, MMNK-1_LIN28B cells also formed larger tumours than control MMNK-1 cells (Fig. 2g, h). Thus, LIN28B promotes cholangiocyte transformation and increases its stemness and growth rate.

### LIN28B promotes the migration and invasion of cholangiocytes

**Fig. 3:**
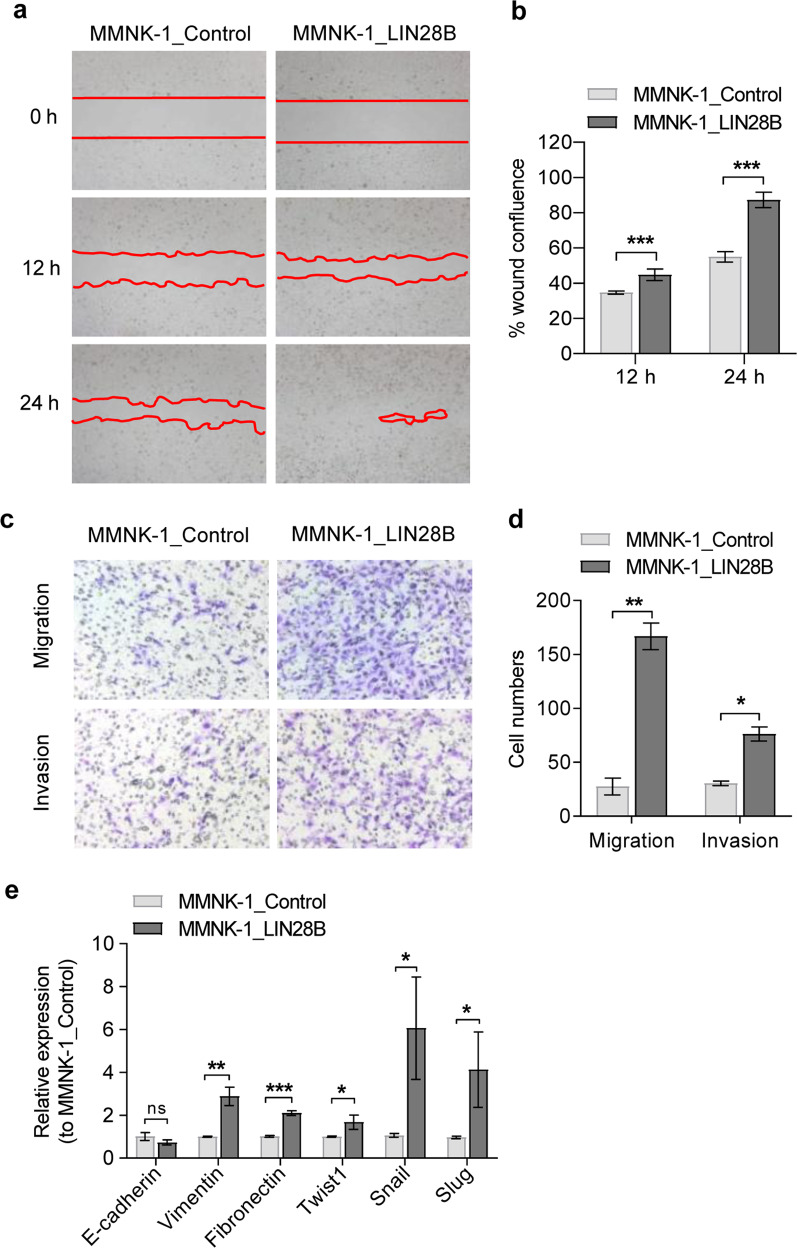
LIN28B promotes epithelial–mesenchymal transition in MMNK-1 cells. The images show control and LIN28B at 0, 12 and 24 h after scratching. The red line indicates the wound gap and shows the line drawn for image analysis (**a**). The percentage of wound confluence was normalised to the starting point, 0 h (**b**). Quantification of cell migration and invasion was performed using a Boyden chamber assay. After incubation for 20 h, representative images of cells stained with crystal violet are shown for migrated and invaded cells (**c**), and the cell numbers were quantified (**d**). Relative expression of the epithelial and mesenchymal genes E-cadherin, Vimentin, Fibronectin, Twist1, Snail and Slug in LIN28B-overexpressing cells (**e**). Bar graph indicated as the mean ± SD, *n* = 3. Statistical significance is indicated by *P < 0.05; **P < 0.01; ***P < 0.001.

Several lines of evidence suggest that EMT plays an important role in CCA progression [22]. EMT is strongly linked with the acquisition of stemness, metastasis and drug resistance. LIN28B overexpression has been reported to induce EMT changes in breast cancer cell lines [23]. We therefore studied whether LIN28B was sufficient to promote EMT changes in cholangiocytes. In the wound closure assay, LIN28B-overexpressing MMNK-1 cells had a significantly higher percentage of wound confluence than the control cells (Fig. 3a, b). In addition, Transwell migration and invasion assay results confirmed the superior migration and invasion capacity of LIN28B-overexpressing MMNK-1 cells over control MNNK-1 cells (Fig. 3c, d). Next, we measured the expression of EMT-related genes using RT-qPCR. As shown in Fig. 3e, the epithelial marker E-cadherin was slightly decreased, whereas the mesenchymal markers vimentin, fibronectin, Twist1, Slug and Snail were mainly upregulated in LIN28B-overexpressing cells compared to control cells. Thus, these data suggest that LIN28B promotes EMT changes in cholangiocytes.

### LIN28B enhances the TGF-β signalling pathway and promotes TGFBI expression

**Fig. 4:**
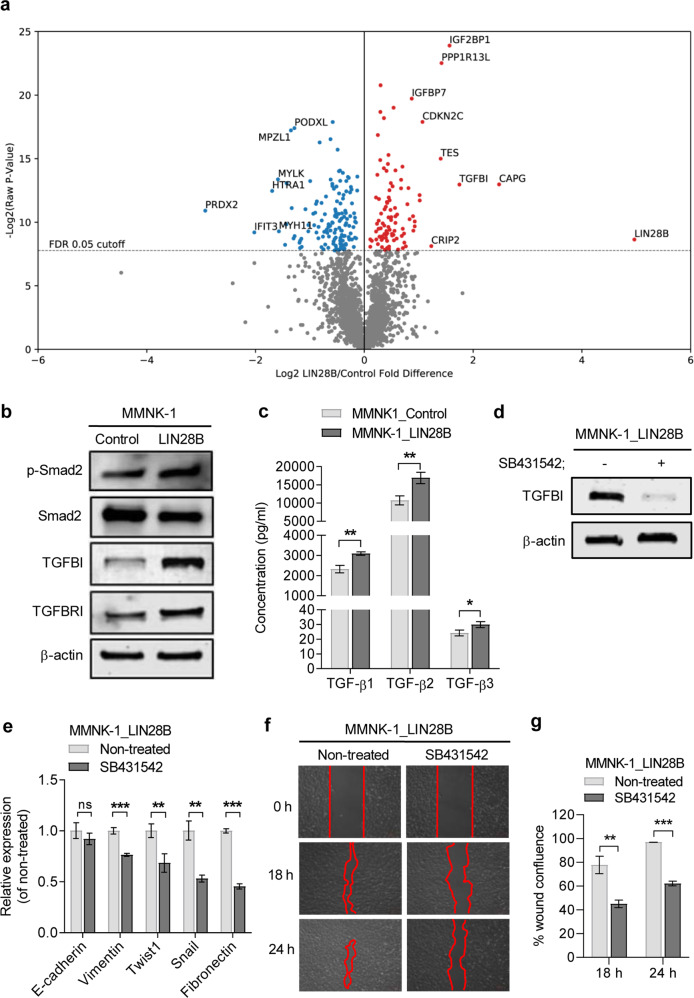
LIN28B enhances components of the TGF-β signalling pathway in MMNK-1 cells. The volcano plot represents the identified proteins and is categorised into upregulated and downregulated expression according to fold change when normalised to control cells (**a**). A false discovery rate (FDR) of 0.05 was used as a cut-off value to consider significant differentially expressed proteins. The expression levels of TGFBI and TGFBRI proteins in control and LIN28B-overexpressing MMNK-1 cells were measured by western blot (**b**). The amount of secreted TGF-β complex in culture medium was measured using Bio-Plex Pro™ TGF-β assays (**c**). TGFBI expression levels in LIN28B-overexpressing MMNK-1 cells treated with the TGFBRI inhibitor SB431542 (10 µM) for 24 h were determined by western blot (**d**). The expression of the indicated genes in LIN28B-overexpressing MMNK-1-treated cells was determined using RT-qPCR, and the data are shown as relative expression to nontreated cells (**e**). The cell migration ability of LIN28B-overexpressing MMNK-1 cells treated with SB431542 was determined by wound healing assay (**f**), and quantitative wound closure was demonstrated by the percentage of wound confluence (**g**). Data are shown as the mean ± SD, *n* = 3. Statistical significance is indicated by *P < 0.05, **P < 0.01, ***P < 0.001.

To elucidate the mechanisms by which LIN28B promotes cholangiocyte transformation, proteomic analysis was conducted to identify differentially expressed proteins in LIN28B-overexpressing MMNK-1 cells and control MMNK-1 cells. Proteomics analysis revealed 234 differentially expressed proteins at a target false discovery rate of 5% (Benjamini-Hochberg correction, *p*-value cut-off = 0.0046). Ninety-five upregulated proteins, including CAPG, IGF2BP1, PP1R13L, and transforming growth factor beta induced protein (TGFBI), and 139 downregulated proteins, including PRDX2, were identified (Fig. 4a). Because TGFBI, an extracellular matrix (ECM) protein involved in cancer metastasis, is a well-known downstream target of the TGF-β pathway [24], we then studied the effect of LIN28B on other components of the TGF-β signalling pathway. Western blot analysis confirmed that TGFBI was upregulated in LIN28B-overexpressing MMNK-1 cells, as well as TGF-β receptor 1 (TGFBRI) (Fig. 4b). Bioplex assays demonstrated that LIN28B-expressing cells secreted significantly higher levels of TGF-β1, β2 and β3 (Fig. 4c). Importantly, treatment with the selective TGF-β inhibitor SB431542 reduced the LIN28B effect on TGFBI expression (Fig. 4d) as well as EMT genes and migratory capability (Fig. 4e–g). These data suggest that LIN28B expression enhances the TGF-β signalling feedback loop and that this interaction contributes to CSC induction and EMT promotion of LIN28B.

### LIN28B promotes cell migration and spheroid formation capability in cholangiocarcinoma cell lines

**Fig. 5:**
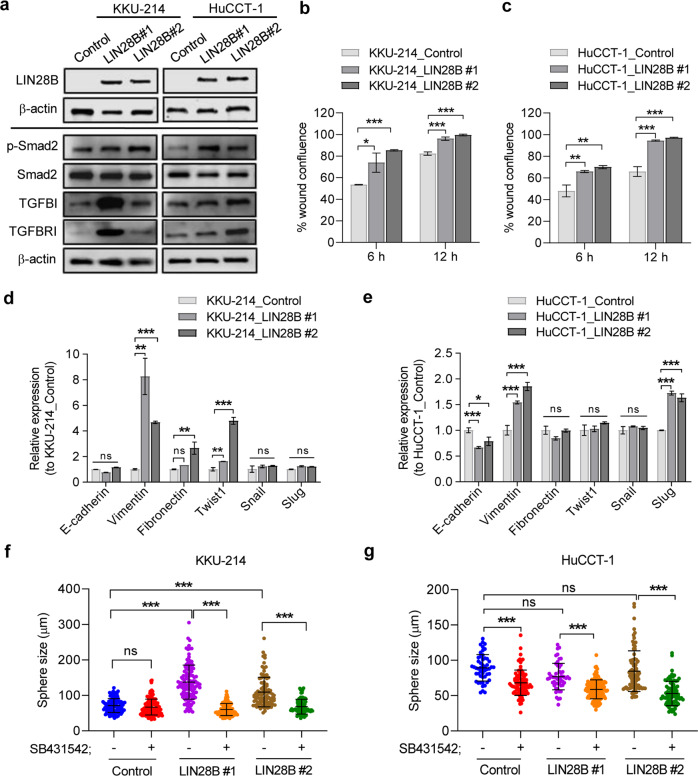
LIN28B enhances the TGF-β pathway and promotes cell migration and sphere-forming capacity in CCA cell lines. Quantitative LIN28B gene expression in two clones of LIN28B-overexpressing CCA cells, #1 and #2, was determined by RT-qPCR (**a**). Data are shown as the fold change of gene expression normalised to cells containing an empty vector control. A cell scratching assay was performed to determine the capability of cells to close the wound gap. Representative images of wound closure of control cells and two clones of LIN28B-overexpressing cells after 6 h and 12 h incubation are shown (**b**). The capability of LIN28B-induced cell migration in KKU-214 and HuCCT-1 cells is shown by the percentage of wound area normalised to the initial wound gap (0 h) (**b**, **c**). The expression of EMT-related genes in LIN28B-overexpressing KKU-214 and HuCCT-1 cells was analysed by RT-qPCR (**d**, **e**). The total number and average size of counting spheroids in control cells and LIN28B-overexpressing KKU-214 (**f**) and HuCCT-1 cells (**g**) are shown as the mean ± SD of triplicate wells. Statistical significance is indicated by **P < 0.01, ***P < 0.001. ns is noted as no statistically significant.

To investigate whether LIN28B can further enhance the aggressive characteristics of cholangiocarcinoma cell lines, we generated stable LIN28B-overexpressing HuCCT-1 and KKU-214 cell lines and analysed their properties (Fig. 5a). All LIN28B-overexpressing cell lines tested (KKU-214_LIN28B and HuCCT-1_LIN28B) demonstrated increased cell migration capability in the wound closure assay compared to parent cell lines (Fig. 5b, c and Supplementary Fig. 2a, b). Real-time PCR showed an increase in the expression of the EMT marker vimentin, although EMT-related transcription factor upregulation seemed to be cell type specific (Fig. 5d, e). Interestingly, LIN28B increased both the number and average size of spheres in KKU-214 (Fig. 5f and Supplementary Fig. 2c) but not in HuCCT-1 cells (Fig. 5g and Supplementary Fig. 2d), implying that the CSC-promoting effect of LIN28B may require supporting factors in certain cell types. Importantly, SB431542 significantly reduced spheroid formation in both KKU-214_LIN28B and HuCCT1-LIN28B cells. Taken together, these results support the model in which enhanced TGF-β signalling by LIN28B promotes cancer stem cell-like potential in CCA.

### TGFBI enhances migration of MMNK-1 and HuCCT-1 cells

**Fig. 6:**
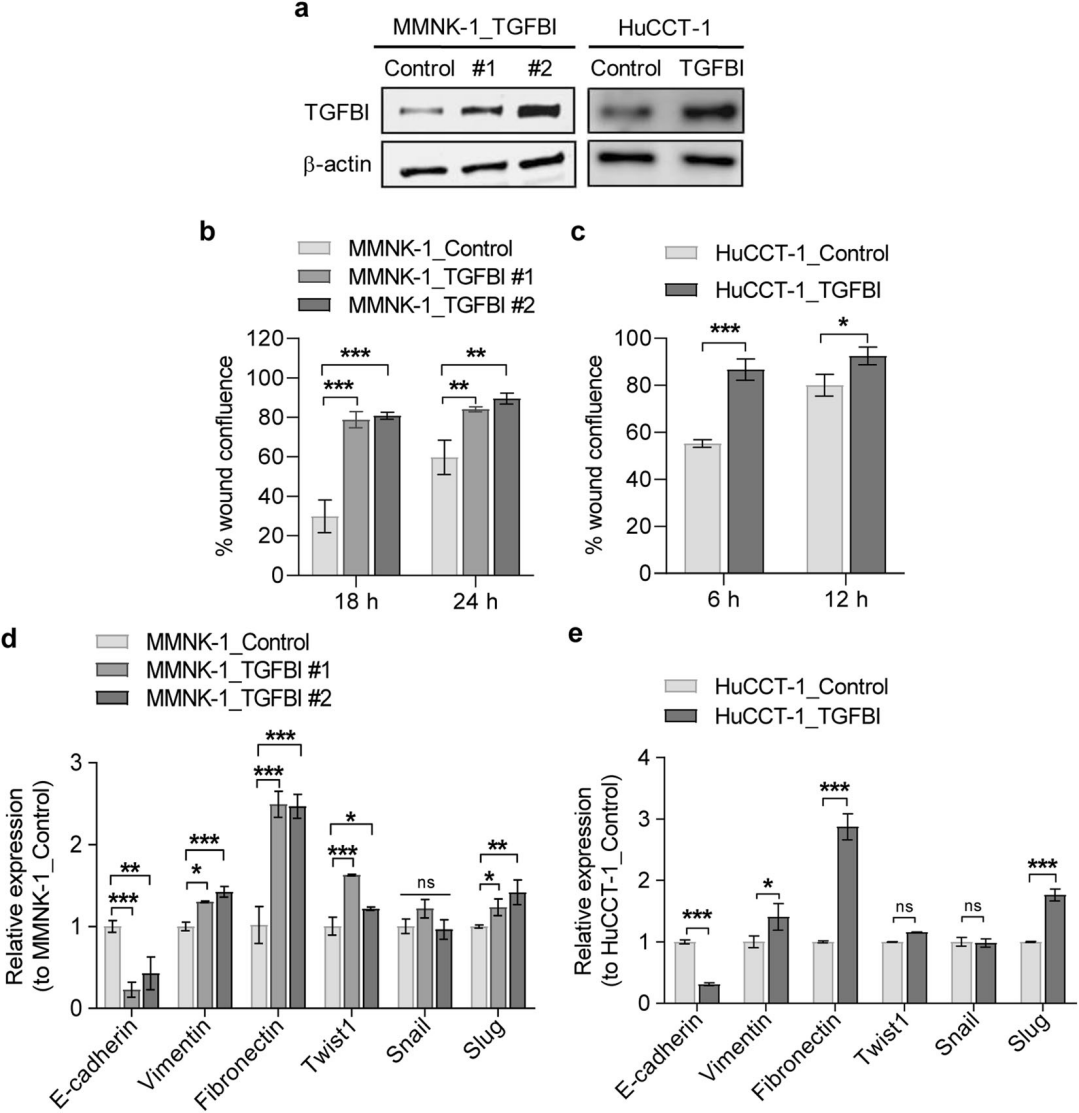
TGFBI induces migration in MMNK-1 and HuCCT-1 cells. The overexpression of exogenic TGFBI protein in MMNK-1 and HuCCT-1 cell lines was detected by western blot (**a**). The effects of TGFBI on MMNK-1 and HuCCT-1 cell migration were evaluated by a cell scratching assay. The efficiency of wound closure was demonstrated in the percentage of wound confluence, normalised to the initial wound area at 0 h (**b**, **c**). The expression of EMT-related genes were determined by RT-qPCR. Data are shown as the fold change of gene expression normalised to control cells (**d**, **e**). Statistical significance is indicated by **P < 0.01, ***P < 0.001.

TGFBI has been reported to be both a tumour promoter and suppressor. To evaluate the effects of TGFBI on normal and CCA cells, we confirmed stable expression of TGFBI in MMNK-1 and HuCCT-1 cells (Fig. 6a). We further performed a cell scratching assay to determine whether TGFBI could promote the cell migration capability of MMNK-1 and HuCCT-1 cells. TGFBI noticeably increased the migration of MMNK-1 cells (Fig. 6b and Supplementary Fig. 3a) and HuCCT-1 cells (Fig. 6c and Supplementary Fig. 3b). Real-time PCR showed that TGFBI enhanced the expression of EMT-related genes in both MMNK-1 and HuCCT-1 cells (Fig. 6d, e). It should be noted that TGFBI overexpression in both MMNK-1 and HuCCT-1 cells was insufficient to increase spheroid-forming capability and sphere size (Supplementary Fig. 4a, b). This indicates that the effect of LIN28B in promoting the clonogenic potential of CCA is likely mediated through other mechanisms.

## Discussion

Consistent with what has been previously reported in other cancer cell lines, such as for breast, colorectal and pancreatic cancers [25–27], LIN28B overexpression in immortalised cholangiocyte and cholangiocarcinoma cell lines enhanced cell proliferation, migratory capacity and clonogenic potential, suggesting that LIN28B is capable of promoting cancer stem cell-like properties in transformed bile duct epithelium. We demonstrated that part of this observation is due to LIN28B enhancing the TGF-β/EMT programme. However, other mechanisms downstream of LIN28B could also play an important role. LIN28B was shown to be capable of activating other stem cell-related gene and stem-like gene networks in prostate cancer [28]. Because not all LIN28B-overexpressing cells formed spheres in our study, it is possible that other factors, such as heterogeneity in epigenetic status or variability in the level of ECM protein expression within the cell population, also contributed to the CSC phenotype observed in this assay.

Although we observed LIN28B expression in only a small subpopulation of cancer cells, reactivation of LIN28B may account for intratumour functional heterogeneity within CCA, and a positive subset of CCA is likely to have more metastatic potential. This model is similar to what was recently described in a study of pancreatic adenocarcinoma (PDAC), in which an unbiased screen identified LIN28B as a signature of small subpopulation circulating tumour cells with high metastasis propensity and most correlate with poor clinical outcome even when LIN28B is expressed in only a small subpopulation of primary PDAC tumours [29]. Nevertheless, it would be important to evaluate the expression of LIN28B in a larger population of cholangiocarcinoma tumours to clearly define the histologic pattern of expression.

It is still unclear whether LIN28B alone is sufficient to induce cancer transformation of normal adult cholangiocytes. In a murine model, LIN28B overexpression in hepatocytes was sufficient to cause hepatoblastoma but only when LIN28B was induced throughout the developmental stage [13]. It is possible that LIN28B could enhance cancer transformation in premalignant cells from other insults. The effect of LIN28B on the expression level of PPP1R3 L, an inhibitory member of the apoptosis-stimulating protein of p53 family (IASPP) [30], as observed in our proteomic study, may also contribute to an increased chance of cancer transformation after genotoxicity.

The interaction between LIN28, let-7 and the TGF-β pathway has been reported to play important roles during foetal tissue development, such as foetal regulatory T cell differentiation [31] and endothelial-to-mesenchymal transition [32]. TGF-β can stimulate LIN28B expression through inhibition of let-7 and direct stimulation at SMAD binding [33], whereas TGFBRI and TGF-β1 are known let-7 targets [34]. In cancer, TGF-β-mediated activation of LIN28B has been reported in PDAC [35]. Our results suggest that this interaction may play a role during CCA metastasis. In cancer, TGF-β acts as a double-edge sword to either limit tumour cell growth by promoting cell cycle exit or enhancing cancer aggressiveness, and cancer stem cell-like properties depend on the cellular context [23, 36, 37]. LIN28B activation could bias TGF-β towards migratory and CSC-like phenotypes by enhancing the expression of TGF-β targets and the TGF-β/let-7/LIN28/TGF-β/TGFBRI positive feedback loop without cell cycle arrest. Since both aberrantly expressed LIN28B and a high TGFBI tumour microenvironment are related to a more advanced disease stage among multiple tumour types, this interaction may also be important for LIN28B-positive CSC-like cells in other tissues. Target components of this feedback loop may provide an opportunity to reduce CSCs, which has been proposed to be responsible for resistance to therapy and relapse.

Our work provides evidence that TGFBI activated by the TGF-β/LIN28B feedback loop promotes CCA aggressiveness. TGFBI was previously shown to be overexpressed in intrahepatic cholangiocarcinoma and associated with areas with high EMT changes [38]. Similar findings were observed in many other types of gastrointestinal cancers [39]. While TGFBI overexpression in CCA cell lines was insufficient to promote clonogenic potential and cell proliferation, it clearly enhanced cell migration. TGFBI has been shown to promote EMT by an unknown mechanism [39]. Recent studies demonstrated that TGFBI also enhances hypoxia and glycolysis in cells in the tumour microenvironment [40, 41] and mediates the immune tolerance of CSCs [42], which could be crucial for CSC maintenance and metastasis. On the other hand, it has been shown that reduction of TGFBI in the tumour microenvironment by downregulation of TGFBI using siRNA or blocking monoclonal antibody can reduce tumour aggressiveness [43, 44]. Together, growing lines of evidence indicate that TGFBI has the potential to serve as a new target for CCA therapeutic strategies in addition to direct inhibition of the TGF-β pathway. Moreover, it should be noted that CAPG, another LIN28B-induced matrix protein identified in our study, has been reported as capable of inducing EMT and promoting tumorigenesis in bladder cancer [45]. Whether CAPG is involved in CCA pathogenesis or CSC maintenance requires further investigation.

## Materials and methods

### Cell lines and cell culture

The human immortalised cholangiocyte cell line MMNK-1, a representative normal bile duct cell line was established as previously described [46], and bile duct cancer HuCCT-1 and KKU-214 cells were obtained from the JCRB Cell Bank, Japan. The HEK293gp cell line was obtained from Stem Cell and Cell Therapy Research Center, Chulalongkorn University, Thailand. MMNK-1, HEK293gp and KKU-214 cells were cultured in high glucose Dulbecco’s modified Eagle’s medium (DMEM) supplemented with 10% FBS, 1% GlutaMAX and 1% antimycotic–antibiotic reagents (Gibco). HuCCT-1 cells were cultured in RPMI 1640 medium (HyClone) supplemented with 10% FBS, 1% GlutaMAX and 1% antimycotic–antibiotic reagents. All cells were maintained at 37 °C in a humidified 5% CO_2_ atmosphere.

### Stable LIN28B-expressing cell lines

The pBabe-control and pBabe-LIN28B constructs were kindly gifted from the Daley Lab. The VSVG plasmid was obtained from the Stem Cell and Cell Therapy Research Unit, Chulalongkorn University, Thailand. For viral production, the compositions of transfection complex formation were prepared following the manufacturer’s protocol. Briefly, the plasmids encoding LIN28B, VSVG, and X-tremeGENE HP DNA transfection reagent (Roche) were diluted with Opti-MEM I medium. HEK293gp cells were incubated with transfection complex for 24 h, and virus was collected after three days of transfection. The cells were incubated with polybrene (Sigma–Aldrich) and virus containing LIN28B and the control. LIN28B and control-expressing cell lines were selected and maintained in medium containing 1 µg/ml puromycin.

### RNA extraction and quantitative real-time PCR

Total RNA was extracted using TRIzol reagent (Ambion). mRNA was reverse transcribed into cDNA using a RevertAidH Minus Kit (Thermo Scientific), and qPCR was performed using SYBR Green master using the set of primers described in Supplementary Table 1. For quantitative analysis of miRNA, cDNA was synthesised using a miScript II RT Kit (Qiagen). The expression of miRNAs was evaluated using miScript Primer Assays (Qiagen) together with the miScript SYBR Green PCR Kit (Qiagen). The relative miRNA and mRNA expression levels were normalised to those of the internal controls, U6 for miRNA and GAPDH for mRNA. The primer sequences are provided in Supplementary Table 1.

### Immunohistochemical staining

Paraffin-embedded CCA tissues were obtained with informed consent from 20 CCA patients who received surgical resection at KCMH, Chulalongkorn University, between 2016-2020. The use of tissues for this study was approved by the Ethics Committee of the Faculty of Medicine, Chulalongkorn University (No. 244/58). Paraffin-embedded sections of CCA were deparaffinized in xylene and rehydrated by submerging in stepwise decreasing concentrations of ethanol. The sections were boiled under pressure in 0.01 M citrate buffer pH 6.0 for antigen unmasking and then incubated with 0.3% H_2_O_2_ in methanol to neutralise endogenous peroxidase. Sections were then submerged in diluted 5% FBS in PBS to block nonspecific binding, incubated with primary antibody dilution, 1:500 of LIN28B (Cell Signaling Technology) overnight before incubation with secondary antibody conjugated HRP (Dako). Peroxidase activity was determined by adding 3,3-diaminobenzidine (DAB) substrate solution (DAKO) and finally counterstained with Mayer’s haematoxylin. Immunofluorescence in CCA tissues was performed using an Opal™ staining Kit (PerkinElmer).

### Western blot

Protein expression was performed by following standard western blotting procedures. Briefly, cellular proteins were extracted from cells using RIPA buffer (Cell Signaling Technology) containing 1X protease and phosphatase inhibitors, and the protein concentration was measured using BCA reagent (Thermo Scientific). Protein was separated with 4–20% Tris-glycine gradient SDS polyacrylamide gel electrophoresis (Bio-Rad) and then transferred onto a nitrocellulose membrane. The membrane was blocked for nonspecific binding in 5% BSA in TBST buffer and subsequently incubated with the following diluted primary antibodies: anti-LIN28B (D4H1) (1:5000, Cell Signaling Technology), total SMAD2 (D43B4) (1:2000, Cell Signaling Technology), phosphoSMAD2 (Ser465/467) (1:1000, Merck Millipore), TGFBRI (H-100) (1:2000, Santa Cruz), TGFBI (1:2000, Abcam), and β-actin (13E5) (1:2000, Cell Signalling Technology) (Supplementary Table 2) overnight at 4 °C. Then, the membrane was incubated for 1 h with the secondary antibody. The level of protein expression was visualised using an Odyssey® CLx imaging system (Li-cor).

### Cell viability assay

In brief, cells were seeded onto 96-well plates, and the medium was removed at the indicated time. Cells were incubated with 0.5 mg/ml MTT (Sigma–Aldrich) in PBS for 3 h at 37 °C. MTT-formazan crystals were solubilized by adding DMSO, and the absorbance was then measured at 570 nm using Varioskan Flash (Thermo Scientific).

### Cell scratching assay

The cells suspended in serum-free media were seeded into each well of a 12-well plate and incubated overnight. Prior to scratching, the medium was removed, a wound was created using a sterile 200 µl pipette tip, and the cells were washed twice with PBS before adding 10% FBS-supplemented medium. The initial wound area and wound area at the indicated times of experiments were captured under an AxioObserver inverted microscope (Zeiss), and the wound gap was measured using ZEN2 blue2.3 software (Zeiss). The percentage of wound confluence was calculated by following a previous study [47].

### Transwell invasion and migration assays

For cell invasion, a Transwell insert 8 µm PET membrane (Corning) was coated with 250 µg/ml Matrigel (Corning). A total of 1 × 10^4^ cells suspended in serum-free media were seeded per well into a Matrigel-coated Transwell insert, and medium containing 10% FBS was added to the lower chamber of a 24-well plate. After the indicated incubation time, the invaded cells were fixed with 4% paraformaldehyde, permeabilized with absolute methanol, and then stained with 0.05% crystal violet in PBS. Non-invaded cells were discarded by cotton swabs. The migration assay procedure was performed as mentioned above, differing only where the cells were seeded on top of an uncoated Matrigel Transwell insert.

### Soft agar colony formation assay

In brief, 0.8% base agar was prepared by boiling 0.8 g of agarose in sterile PBS and then mixing with culture medium prior to addition to a six-well plate. A total of 3 × 10^4^ cells were suspended in top agar (1:1 dilution of base agar and culture medium), and 1 ml was added to the top base agar. Cells were cultured for 4 weeks and fed twice per week. Randomised fields of each well were captured under an Axio Observer inverted microscope (Zeiss) with z stack and mosaic programmes. All colonies were counted, and the size of all colonies was measured using ZEN2 blue 2.3 software.

### Spheroid formation

A total of 1.5 × 10^3^ cells were suspended in sphere media consisting of DMEM/F12 supplemented with Gluta max, antibiotic-antimycotic B27, 20 ng/ml human recombinant epidermal growth factor (hEGF) and human basic fibroblast growth factor (hbFGF). Cells were then seeded into ultralow attachment 24-well plates. Cells were cultured for 1 week, and all spheroids were captured under an inverted microscope (ZEISS). Spheroids were counted, and the size was measured by ZEN2 blue 2.3 software. In the inhibitor treatment assay, cells were pre-treated with 10 μM SB431542 inhibitor (Tocris Bioscience) for 24 h and then assayed and continuously cultured in media containing SB431542.

### Xenograft mice

The protocol of the animal study was approved by Chulalongkorn University Animal Care and Use Committee of Chulalongkorn University Laboratory Animal Center (CULAC) and preceded in accordance with CULAC guidelines. Male NSG mice (Jackson Laboratory) (4–6 weeks) were subcutaneously injected into the left and right lower flanks with 100 µl of MMNK-1 control and LIN28B-overexpressing MMNK-1 cell suspension (1 × 10^6^ cells per site) in Matrigel (Corning). All mice were terminated on day 28 of the experiment, and tumour size was measured. Tumour volume was determined using the formula *V* = 0.5 × L × W2 (L = long and W = short tumour diameter).

### TGF-β cytokine array

After culturing cells for 24 h, the medium was collected and centrifuged at 1000×*g* for 15 min at 4 °C, and then the supernatant was collected. Differential concentrations of cytokines were evaluated using Bio-Plex Pro™ Human TGF-β cytokines (Bio-Rad) according to the manufacturer’s instructions. The concentrations of cytokines were analysed relative to the cytokine standard curve.

### Proteomic analysis

#### Protein extraction and in-solution digestion

The cells were lysed with a mixture of 5% sodium deoxycholate (SDC) and 1X protease inhibitor. The protein concentrations were measured by using a BCA protein assay. The sample containing 250 µg of protein was prepared for in-solution digestion methods, following a previous study [48]. Peptides in the MMNK-1_control sample were labelled with light isotope reagents (formaldehyde; CH2O and cyanoborohydride; NaBH3CN), and peptides in the MMNK-1_LIN28B sample were labelled with heavy isotope reagents (13C-labelled formaldehyde; 13CD2O and cyanoborodeuteride; NaBD3CN). The samples were fractionated into 10 fractions using a Pierce High pH Reversed-Phase Peptide Fractionation Kit (Thermo Scientific). All eluted fractions were subjected to LC-MS/MS analyses on a Q Exactive Plus mass spectrometer.

### Data analysis

Raw mass spectra files were analysed with MaxQuant software (version 1.6.2.10) and searched against the Human Swiss-Prot Database (20,408 proteins, downloaded December 2018) as well as a list of common protein contaminants (http://www.thegpm.org). Trypsin/P was set as the digestion enzyme with a maximum of two missed cleavages allowed. Carbamidomethylation of cysteine and acetylation at the N-terminus were set as fixed modifications, while oxidation of methionine was set as a variable modification. Dimethyl labelling was selected as the quantification mode with dimethyl Lys 0 and N-term 0 as the light labels, dimethyl Lys 4 and N-term 4 as the intermediate labels and Lys 8 and N-term 8 as the intermediate labels. The maximum false discovery rate (FDR) was set at 1% for both peptide and protein levels. Other parameters in MaxQuant were set at their default values. Heavy-to-light protein expression ratios were extracted from the protein group output of MaxQuant, log-transformed, and subjected to one-sample Student’s *t* test analyses. The list of 234 significant differentially expressed protein groups was selected at a false discovery rate of 5% using the Benjamini-Hochberg procedure. Only 2539 protein groups with at least 2 razor and unique peptides were considered here. Gene set enrichment analysis was performed using the WebGestalt online interface (https://academic.oup.com/nar/article/47/W1/W199/5494758) with Panther pathway annotation (https://academic.oup.com/nar/article/47/D1/D419/5165346).

### Statistical analysis

Data are displayed as the mean ± SD using GraphPad Prism version 8. For statistical analysis, Student’s *t* test was performed, and *P* values indicated by *P < 0.05, **P < 0.01 and ***P < 0.001 were considered statistically significant.

## Supplementary information

Supplementary figure legends

Supplement Figure 1

Supplement Figure 2

Supplement Figure 3

Supplement Figure 4

Supplement Table 1

Supplement Table 2

qPCR

Western blot

## References

[1] Massarweh NN, El-Serag HB (2017). Epidemiology of hepatocellular carcinoma and intrahepatic cholangiocarcinoma. Cancer Control.

[2] Banales JM, Cardinale V, Carpino G, Marzioni M, Andersen JB, Invernizzi P (2016). Expert consensus document: cholangiocarcinoma: current knowledge and future perspectives consensus statement from the European Network for the Study of Cholangiocarcinoma (ENS-CCA). Nat Rev Gastroenterol Hepatol.

[3] Sripa B, Pairojkul C (2008). Cholangiocarcinoma: lessons from Thailand. Curr Opin Gastroenterol.

[4] Lendvai G, Szekerczes T, Illyes I, Dora R, Kontsek E, Gogl A (2020). Cholangiocarcinoma: classification, histopathology and molecular carcinogenesis. Pathol Oncol Res.

[5] Wu HJ, Chu PY (2019). Role of cancer stem cells in cholangiocarcinoma and therapeutic implications. Int J Mol Sci.

[6] Shyh-Chang N, Daley GQ (2013). Lin28: primal regulator of growth and metabolism in stem cells. Cell Stem Cell.

[7] Viswanathan SR, Powers JT, Einhorn W, Hoshida Y, Ng TL, Toffanin S (2009). Lin28 promotes transformation and is associated with advanced human malignancies. Nat Genet.

[8] Viswanathan SR, Daley GQ, Gregory RI (2008). Selective blockade of microRNA processing by Lin28. Science.

[9] Newman MA, Thomson JM, Hammond SM (2008). Lin-28 interaction with the Let-7 precursor loop mediates regulated microRNA processing. RNA.

[10] Heo I, Joo C, Cho J, Ha M, Han J, Kim VN (2008). Lin28 mediates the terminal uridylation of let-7 precursor MicroRNA. Mol Cell.

[11] Wilbert ML, Huelga SC, Kapeli K, Stark TJ, Liang TY, Chen SX (2012). LIN28 binds messenger RNAs at GGAGA motifs and regulates splicing factor abundance. Mol Cell.

[12] Viswanathan SR, Daley GQ (2010). Lin28: a microRNA regulator with a macro role. Cell.

[13] Nguyen LH, Robinton DA, Seligson MT, Wu L, Li L, Rakheja D (2014). Lin28b is sufficient to drive liver cancer and necessary for its maintenance in murine models. Cancer Cell.

[14] Zhou J, Ng SB, Chng WJ (2013). LIN28/LIN28B: an emerging oncogenic driver in cancer stem cells. Int J Biochem Cell Biol.

[15] Wang H, Zhao Q, Deng K, Guo X, Xia J (2016). Lin28: an emerging important oncogene connecting several aspects of cancer. Tumour Biol.

[16] Balzeau J, Menezes MR, Cao S, Hagan JP (2017). The LIN28/let-7 pathway in cancer. Front Genet.

[17] Chen L, Yan HX, Yang W, Hu L, Yu LX, Liu Q (2009). The role of microRNA expression pattern in human intrahepatic cholangiocarcinoma. J Hepatol.

[18] Wang M, Wen TF, He LH, Li C, Zhu WJ, Trishul NM (2015). A six-microRNA set as prognostic indicators for bile duct cancer. Int J Clin Exp Med.

[19] Yang H, Li TW, Peng J, Tang X, Ko KS, Xia M (2011). A mouse model of cholestasis-associated cholangiocarcinoma and transcription factors involved in progression. Gastroenterology.

[20] McDaniel K, Hall C, Sato K, Lairmore T, Marzioni M, Glaser S (2016). Lin28 and let-7: roles and regulation in liver diseases. Am J Physiol Gastrointest Liver Physiol.

[21] Guo Y, Chen Y, Ito H, Watanabe A, Ge X, Kodama T (2006). Identification and characterization of lin-28 homolog B (LIN28B) in human hepatocellular carcinoma. Gene.

[22] Vaquero J, Guedj N, Claperon A, Nguyen Ho-Bouldoires TH, Paradis V, Fouassier L (2017). Epithelial-mesenchymal transition in cholangiocarcinoma: From clinical evidence to regulatory networks. J Hepatol.

[23] Liu Y, Li H, Feng J, Cui X, Huang W, Li Y (2013). Lin28 induces epithelial-to-mesenchymal transition and stemness via downregulation of let-7a in breast cancer cells. PLoS ONE.

[24] Thapa N, Lee BH, Kim IS (2007). TGFBIp/betaig-h3 protein: a versatile matrix molecule induced by TGF-beta. Int J Biochem Cell Biol.

[25] Xiong H, Zhao W, Wang J, Seifer BJ, Ye C, Chen Y (2017). Oncogenic mechanisms of Lin28 in breast cancer: new functions and therapeutic opportunities. Oncotarget.

[26] King CE, Wang L, Winograd R, Madison BB, Mongroo PS, Johnstone CN (2011). LIN28B fosters colon cancer migration, invasion and transformation through let-7-dependent and -independent mechanisms. Oncogene.

[27] Kugel S, Sebastian C, Fitamant J, Ross KN, Saha SK, Jain E (2016). SIRT6 suppresses pancreatic cancer through control of Lin28b. Cell.

[28] Lovnicki J, Gan Y, Feng T, Li Y, Xie N, Ho CH (2020). LIN28B promotes the development of neuroendocrine prostate cancer. J Clin Invest.

[29] Franses JW, Philipp J, Missios P, Bhan I, Liu A, Yashaswini C (2020). Pancreatic circulating tumor cell profiling identifies LIN28B as a metastasis driver and drug target. Nat Commun.

[30] Bergamaschi D, Samuels Y, O’Neil NJ, Trigiante G, Crook T, Hsieh JK (2003). iASPP oncoprotein is a key inhibitor of p53 conserved from worm to human. Nat Genet.

[31] Bronevetsky Y, Burt TD, McCune JM (2016). Lin28b regulates fetal regulatory t cell differentiation through modulation of TGF-beta signaling. J Immunol.

[32] Chen PY, Qin L, Barnes C, Charisse K, Yi T, Zhang X (2012). FGF regulates TGF-beta signaling and endothelial-to-mesenchymal transition via control of let-7 miRNA expression. Cell Rep.

[33] Park JT, Kato M, Lanting L, Castro N, Nam BY, Wang M (2014). Repression of let-7 by transforming growth factor-beta1-induced Lin28 upregulates collagen expression in glomerular mesangial cells under diabetic conditions. Am J Physiol Ren Physiol.

[34] Zhang Z, Zhang S, Ma P, Jing Y, Peng H, Gao WQ (2015). Lin28B promotes melanoma growth by mediating a microRNA regulatory circuit. Carcinogenesis.

[35] Ottaviani S, Stebbing J, Frampton AE, Zagorac S, Krell J, de Giorgio A (2018). TGF-beta induces miR-100 and miR-125b but blocks let-7a through LIN28B controlling PDAC progression. Nat Commun.

[36] Bellomo C, Caja L, Moustakas A (2016). Transforming growth factor beta as regulator of cancer stemness and metastasis. Br J Cancer.

[37] Wang Y, Li J, Guo S, Ouyang Y, Yin L, Liu S (2017). Lin28B facilitates the progression and metastasis of pancreatic ductal adenocarcinoma. Oncotarget.

[38] Jinawath N, Chamgramol Y, Furukawa Y, Obama K, Tsunoda T, Sripa B (2006). Comparison of gene expression profiles between Opisthorchis viverrini and non-Opisthorchis viverrini associated human intrahepatic cholangiocarcinoma. Hepatology.

[39] Han B, Cai H, Chen Y, Hu B, Luo H, Wu Y (2015). The role of TGFBI (betaig-H3) in gastrointestinal tract tumorigenesis. Mol Cancer.

[40] Costanza B, Rademaker G, Tiamiou A, De Tullio P, Leenders J, Blomme A (2019). Transforming growth factor beta-induced, an extracellular matrix interacting protein, enhances glycolysis and promotes pancreatic cancer cell migration. Int J Cancer.

[41] Fico F, Santamaria-Martinez A (2020). TGFBI modulates tumour hypoxia and promotes breast cancer metastasis. Mol Oncol.

[42] Zhu J, Nie S, Wu J, Lubman DM (2013). Target proteomic profiling of frozen pancreatic CD24+ adenocarcinoma tissues by immuno-laser capture microdissection and nano-LC-MS/MS. J Proteome Res.

[43] Steitz AM, Steffes A, Finkernagel F, Unger A, Sommerfeld L, Jansen JM (2020). Tumor-associated macrophages promote ovarian cancer cell migration by secreting transforming growth factor beta induced (TGFBI) and tenascin C. Cell Death Dis.

[44] Corona A, Blobe GC (2021). The role of the extracellular matrix protein TGFBI in cancer. Cell Signal.

[45] Zhaojie L, Yuchen L, Miao C, Yacun C, Shayi W, Anbang H (2019). Gelsolin-like actin-capping protein has prognostic value and promotes tumorigenesis and epithelial-mesenchymal transition via the Hippo signaling pathway in human bladder cancer. Ther Adv Med Oncol.

[46] Maruyama M, Kobayashi N, Westerman KA, Sakaguchi M, Allain JE, Totsugawa T (2004). Establishment of a highly differentiated immortalized human cholangiocyte cell line with SV40T and hTERT. Transplantation.

[47] Buachan P, Chularojmontri L, Wattanapitayakul SK (2014). Selected activities of Citrus maxima Merr. fruits on human endothelial cells: enhancing cell migration and delaying cellular aging. Nutrients.

[48] Makjaroen J, Somparn P, Hodge K, Poomipak W, Hirankarn N, Pisitkun T (2018). Comprehensive proteomics identification of IFN-lambda3-regulated antiviral proteins in HBV-transfected cells. Mol Cell Proteom.

