# Supplementary material for: The LIN28B/TGF-β/TGFBI feedback loop promotes cell migration and tumour initiation potential in cholangiocarcinoma
Source: Cancer Gene Ther. 2021 Sep 21;29(5):445–55. doi: 10.1038/s41417-021-00387-5 (PMC9113936; doi:10.1038/s41417-021-00387-5)
Supplement: Supplementary file 1 — Supplementary figure legends [file 41417_2021_387_MOESM1_ESM.docx]

**Supplementary Figure Legends**

**Figure S1. Generation of LIN28B-overexpressing MMNK-1 cells.** (a) Western blots showing LIN28B protein expression in MMNK-1_Control and LIN28B-overexpressing MMNK-1 cells. (b) Real-time PCR showing the relative expression of let-7a, let7b and let-7i in LIN28B-overexpressing MMNK-1 cells compared to control cells. ****P*<0.001.

**Figure S2. LIN28B induces cell migration and sphere-forming capacity in CCA cell lines.** (a-b) Representative pictures of cell scratching of LIN28B-overexpressing KKU-214 and HuCCT-1 cells (two cell clones; #1 and #2) after initial time for 6 h and 12 h. (c-d) Representative picture of spheroids of LIN28B-overexpressing KKU-214 and HuCCT-1 cells (two cell clones; #1 and #2) treated with SB431542 inhibitor. Scale bar, 100 μm

**Figure S3. TGFBI induces cell migration.** (a) Representative pictures of the wound closure capacity of TGFBI-overexpressing MMNK-1 cells (two cell clones; #1 and #2) after scratching the cells for 18 h and 24 h and (b) in TGFBI-overexpressing HuCCT-1 cells for 6 h and 12 h.

**Figure S4. Sphere-forming capacity of TGFBI-overexpressing cells.** (a) Representative pictures of spheroids of TGFBI-overexpressing MMNK-1 (two cell clones, #1 and #2) and HuCCT-1 cells. (b) Spheroid size of TGFBI-overexpressing MMNK-1 and HuCCT-1 cells. Scale bar, 100 μm. n = 3; error bars represent the mean±SD. ns stands for not statistically significant.
