# Supplementary figures and images for: The LIN28B/TGF-β/TGFBI feedback loop promotes cell migration and tumour initiation potential in cholangiocarcinoma
Source: Cancer Gene Ther. 2021 Sep 21;29(5):445–55. doi: 10.1038/s41417-021-00387-5 (PMC9113936; doi:10.1038/s41417-021-00387-5)

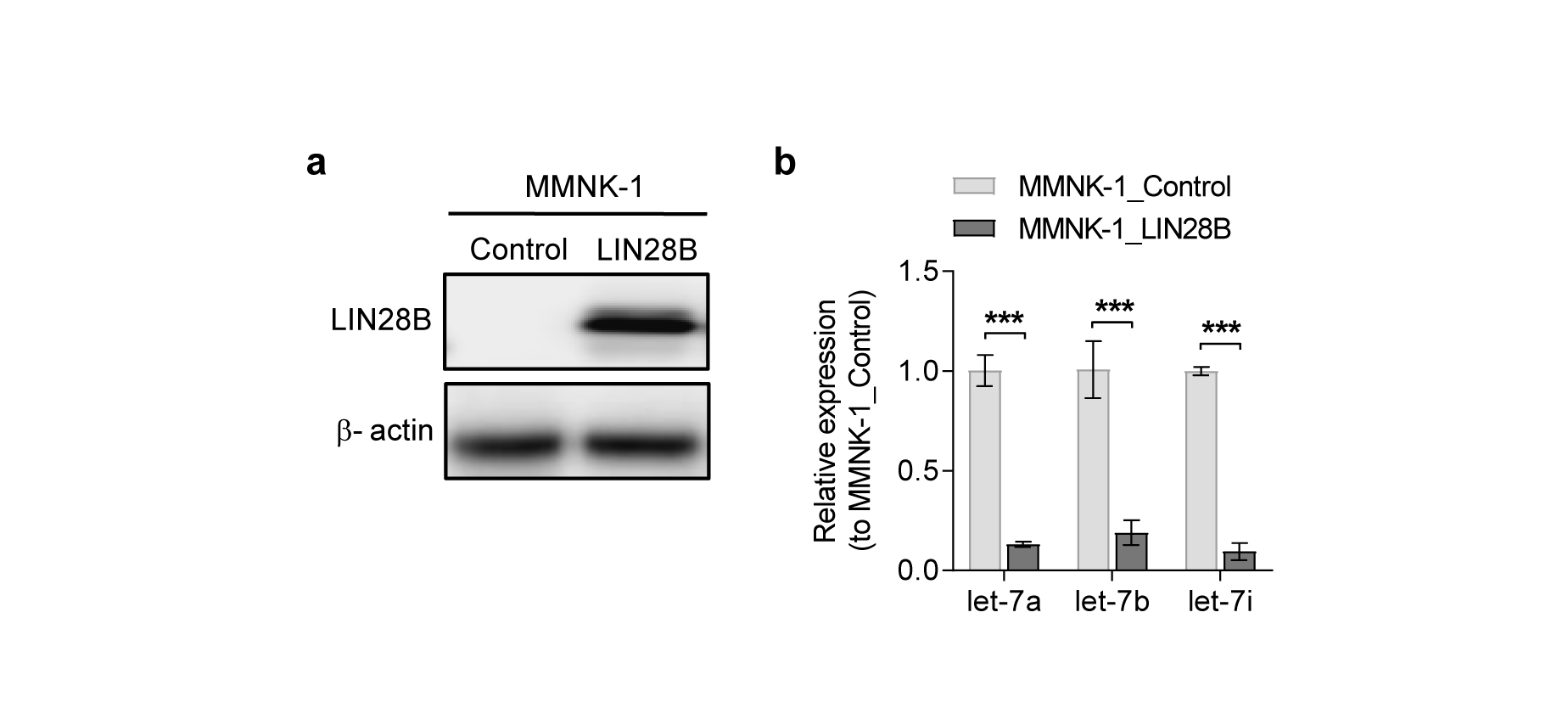

Supplement: Supplementary file 2 — Supplement Figure 1 [file 41417_2021_387_MOESM2_ESM.tif]

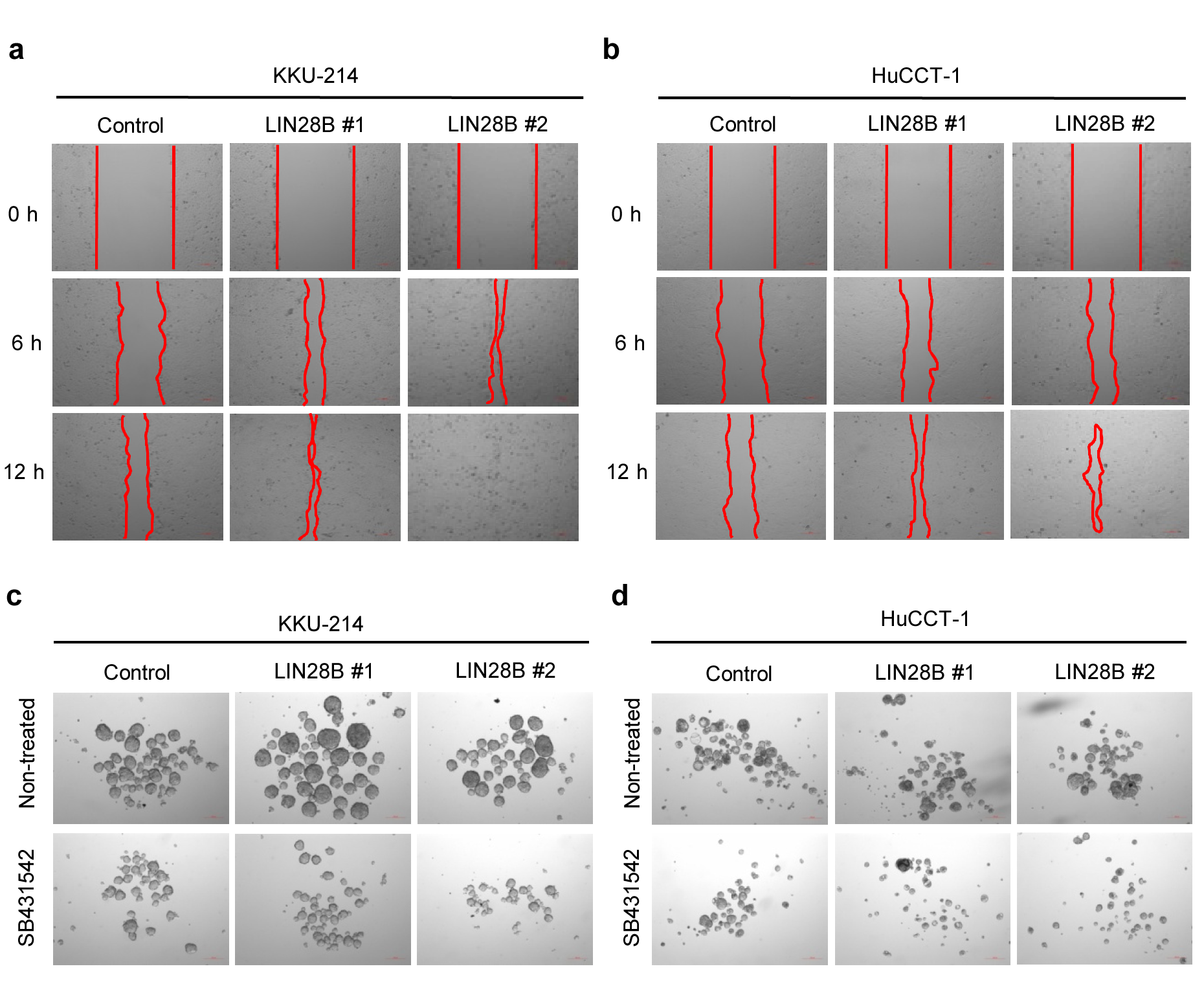

Supplement: Supplementary file 3 — Supplement Figure 2 [file 41417_2021_387_MOESM3_ESM.tif]

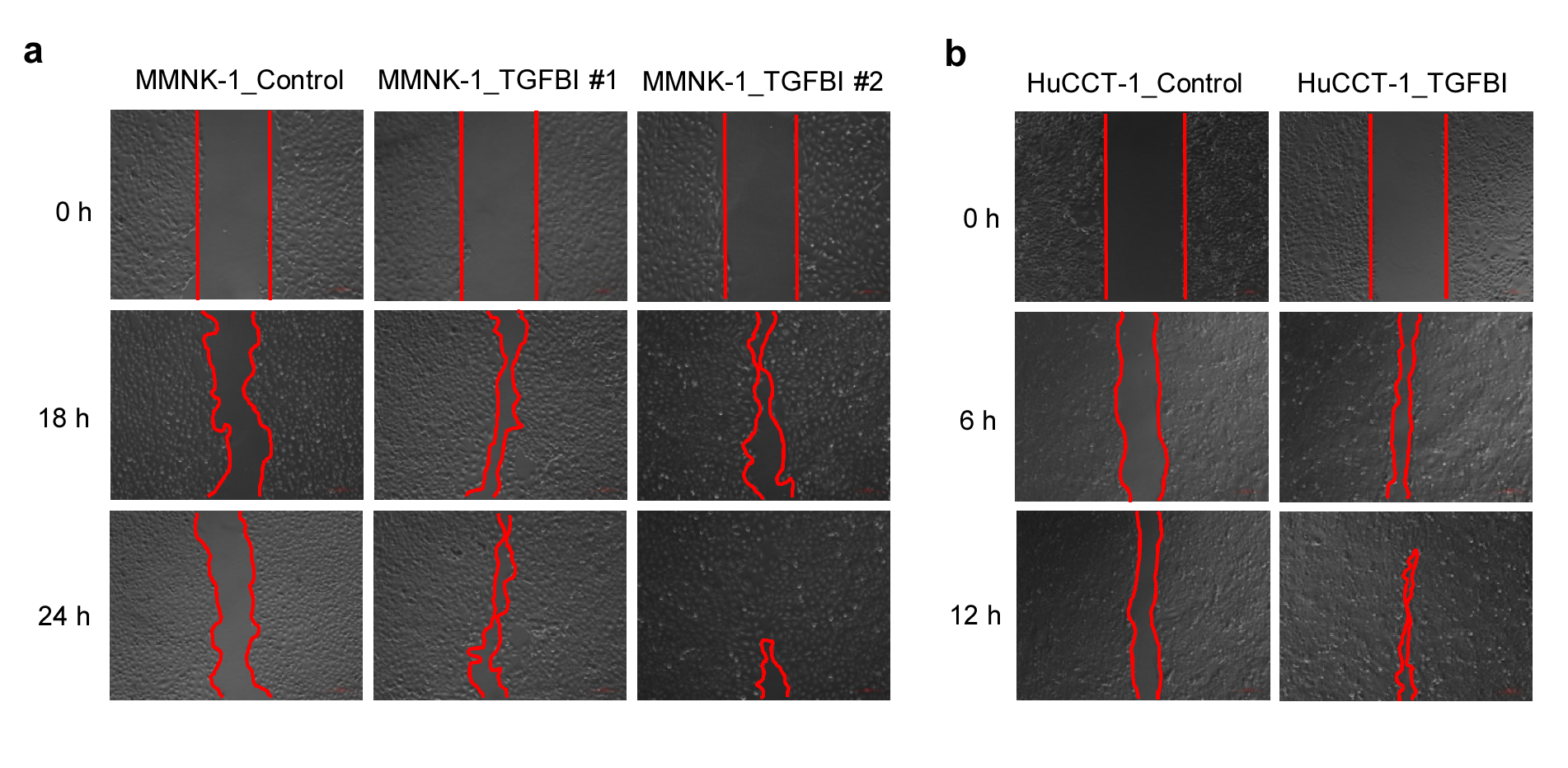

Supplement: Supplementary file 4 — Supplement Figure 3 [file 41417_2021_387_MOESM4_ESM.tif]

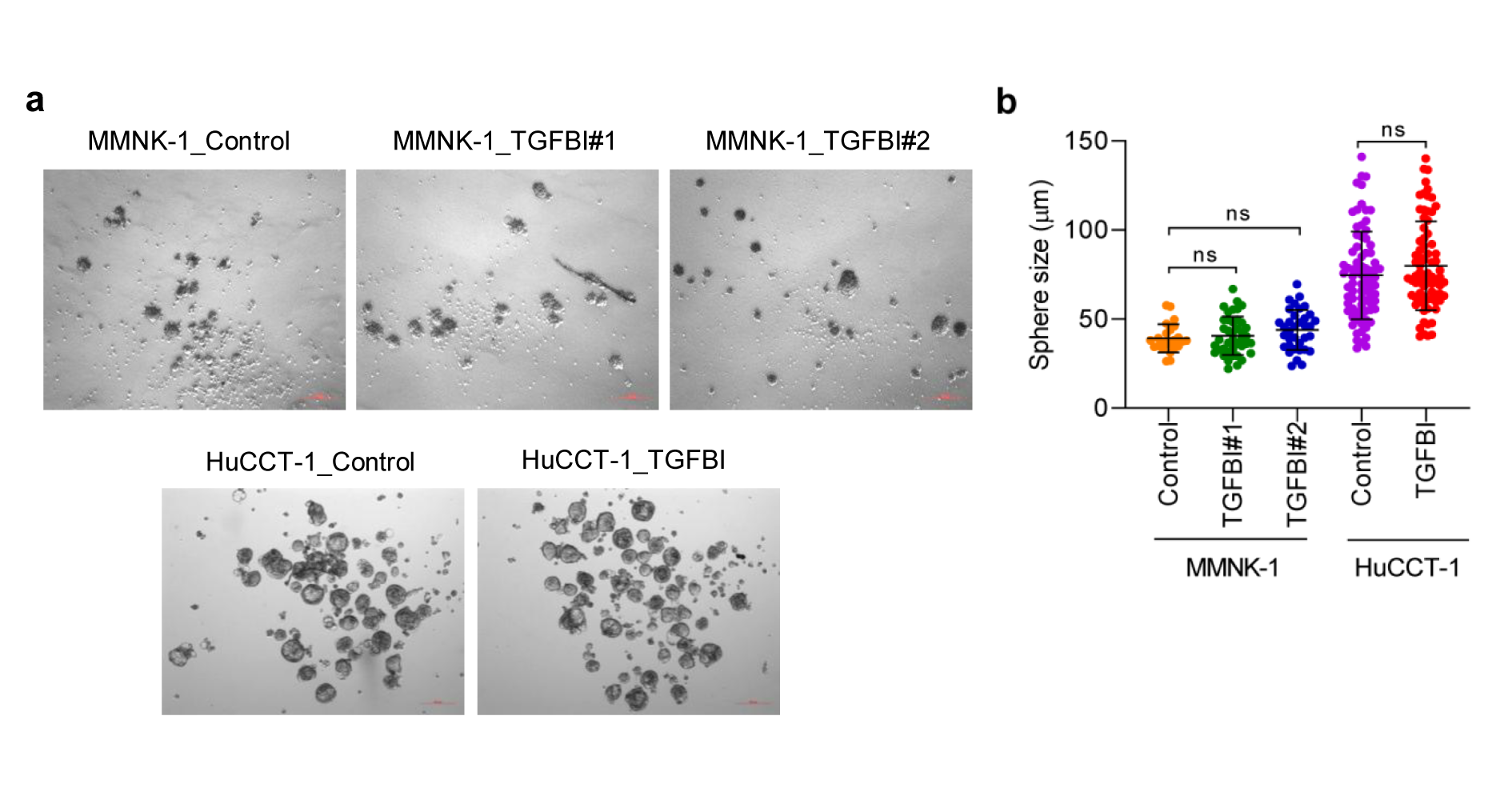

Supplement: Supplementary file 5 — Supplement Figure 4 [file 41417_2021_387_MOESM5_ESM.tif]
