# Supplementary material for: The LIN28B/TGF-β/TGFBI feedback loop promotes cell migration and tumour initiation potential in cholangiocarcinoma
Source: Cancer Gene Ther. 2021 Sep 21;29(5):445–55. doi: 10.1038/s41417-021-00387-5 (PMC9113936; doi:10.1038/s41417-021-00387-5)
Supplement: Supplementary file 6 — Supplement Table 1 [file 41417_2021_387_MOESM6_ESM.docx]

**Supplementary table 1**

Table. List of primers and sequences

| Gene | Forward primer (5'→ 3') | Reverse primer (5'→ 3') |
| --- | --- | --- |
| CCND1 | AACTACCTGGACCGCTTCCT | CCACTTGAGCTTGTTCACCA |
| CDC25A | GAGATCGCCTGGGTAATGAA | TGCGGAACTTCTTCAGGTCT |
| CDK6 | AGAGACAGGAGTGGCCTTGA | TGAAAGCAAGCAAACAGGTG |
| E-cadherin | TGCCCAGAAAATGAAAAAGG | GTGTATGTGGCAATGCGTTC |
| Vimentin | GCAAAGATTCCACTTTGCGT | GAAATTGCAGGAGGAGATGC |
| Fibronectin | CTGTGACAACTGCCGTAG | CAGCTTCTCCAAGCATCG |
| Twist1 | GTCCGCAGTCTTACGAGGAG | CCAGCTTGAGGGTCTGAATC |
| Snail | CACTATGCCGCGCTCTTTC | GGTCGTAGGGCTGCTGGAA |
| Slug | CTTTTTCTTGCCCTCACTGC | GCTTCGGAGTGAAGAAATGC |
| GAPDH | CTGGGCTACACTGAGCACC | AAGTGGTCGTTGAGGGCAATG |
| For miRNAs | | |
| Let-7a | miScript Primer Assays let-7a-specific primer, Qiagen (Cat. no. MS00031220) | |
| Let-7b | miScript Primer Assays let-7b-specific primer, Qiagen (Cat. no. MS00003122) | |
| Let-7i | miScript Primer Assays let-7i-specific primer, Qiagen (Cat. no. MS00003157) | |
| U6 | miScript Primer Assays U6-specific primer, Qiagen (Cat. no. MS00033740) | |
