# Supplementary material for: The LIN28B/TGF-β/TGFBI feedback loop promotes cell migration and tumour initiation potential in cholangiocarcinoma
Source: Cancer Gene Ther. 2021 Sep 21;29(5):445–55. doi: 10.1038/s41417-021-00387-5 (PMC9113936; doi:10.1038/s41417-021-00387-5)
Supplement: Supplementary file 7 — Supplement Table 2 [file 41417_2021_387_MOESM7_ESM.docx]

Supplementary Table 2

| Antibody | Antibody dilution | Cat number and company |
| --- | --- | --- |
| LIN28B (D4H1) | 1: 2000 (WB)  1: 100 (IF) | 11965S, Cell Signaling Technology |
| Cytokeratin 7 (CK7) (EPR1619Y) | 1: 100 (IF) | ab68459, abcam |
| β-actin (13E5) | 1: 2000 (WB) | 4970T, Cell Signaling Technology |
| Smad2 (D43B4) | 1: 2000 (WB) | 5339P, Cell Signaling Technology |
| TGFBRI (H-100) | 1: 2000 (WB) | SC-9048, Santa Cruz |
| TGFBI [EPR12078(B)] | 1: 2000 (WB) | ab170874, abcam |
| phospho-Smad2 (Ser465/467) | 1: 1000 (WB) | AB3849, Merck Millipore |
| Secondary antibody;  [IRDye 800CW Goat anti-Rabbit IgG](https://www.licor.com/bio/reagents/irdye-800cw-goat-anti-rabbit-igg-secondary-antibody) | 1:2000 (WB) | 926-32211, LI-COR |
| Opal 4-Color Manual IHC Kit;  Opal fluorophore | 1: 100 (IF) | NEL810001KT, PerkinElmer |

WB; Western blot, IF; immunofluorescence staining (opal)
