# Supplementary material for: The LIN28B/TGF-β/TGFBI feedback loop promotes cell migration and tumour initiation potential in cholangiocarcinoma
Source: Cancer Gene Ther. 2021 Sep 21;29(5):445–55. doi: 10.1038/s41417-021-00387-5 (PMC9113936; doi:10.1038/s41417-021-00387-5)

Figure 4b, western blot; p-Smad2, Smad2, TGFBI, TGFBRI

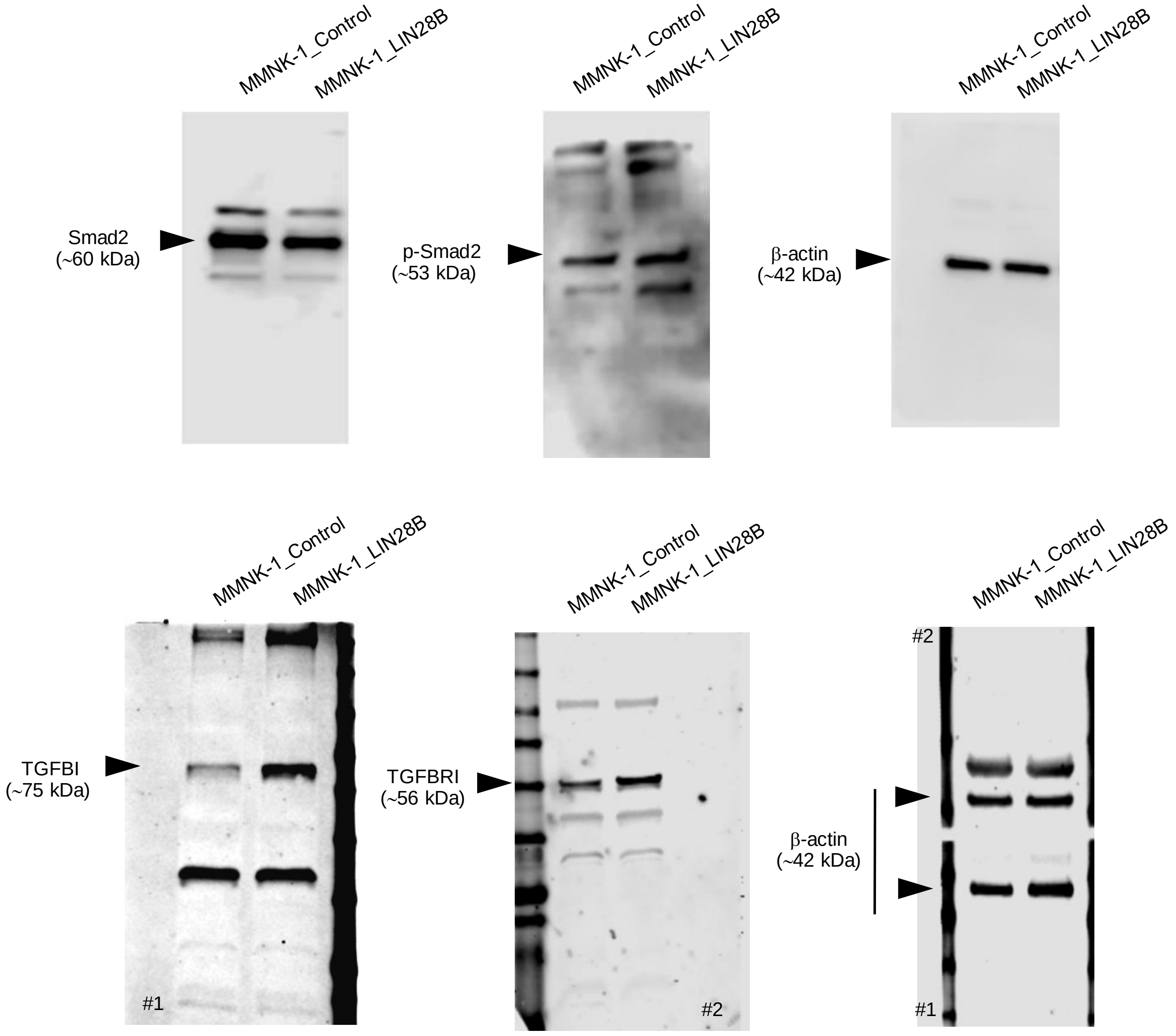

Figure 4d, western blot; TGFBI

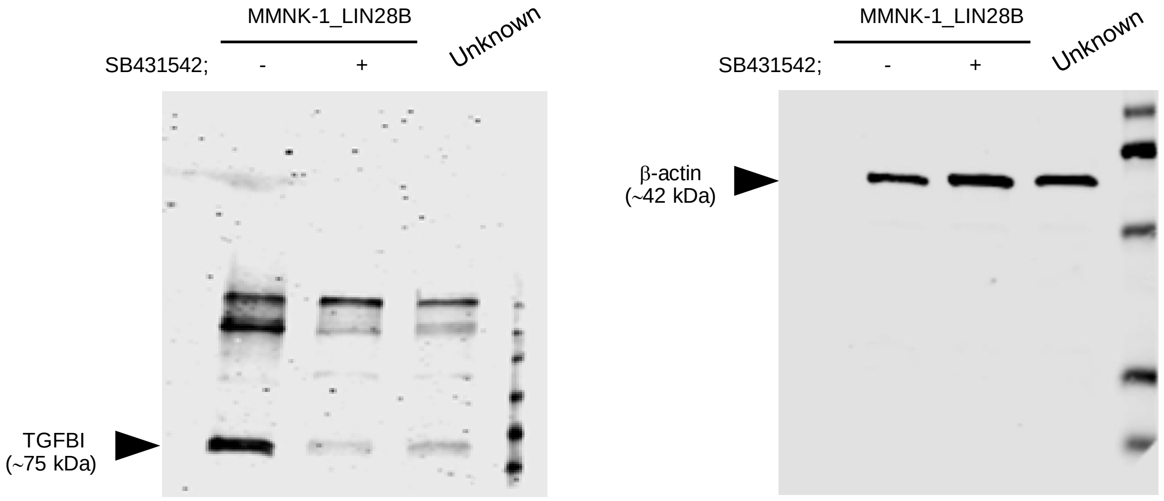

Figure 5a, western blot; LIN28B, Smad2, p-Smad2, TGFBI, TGFBRI

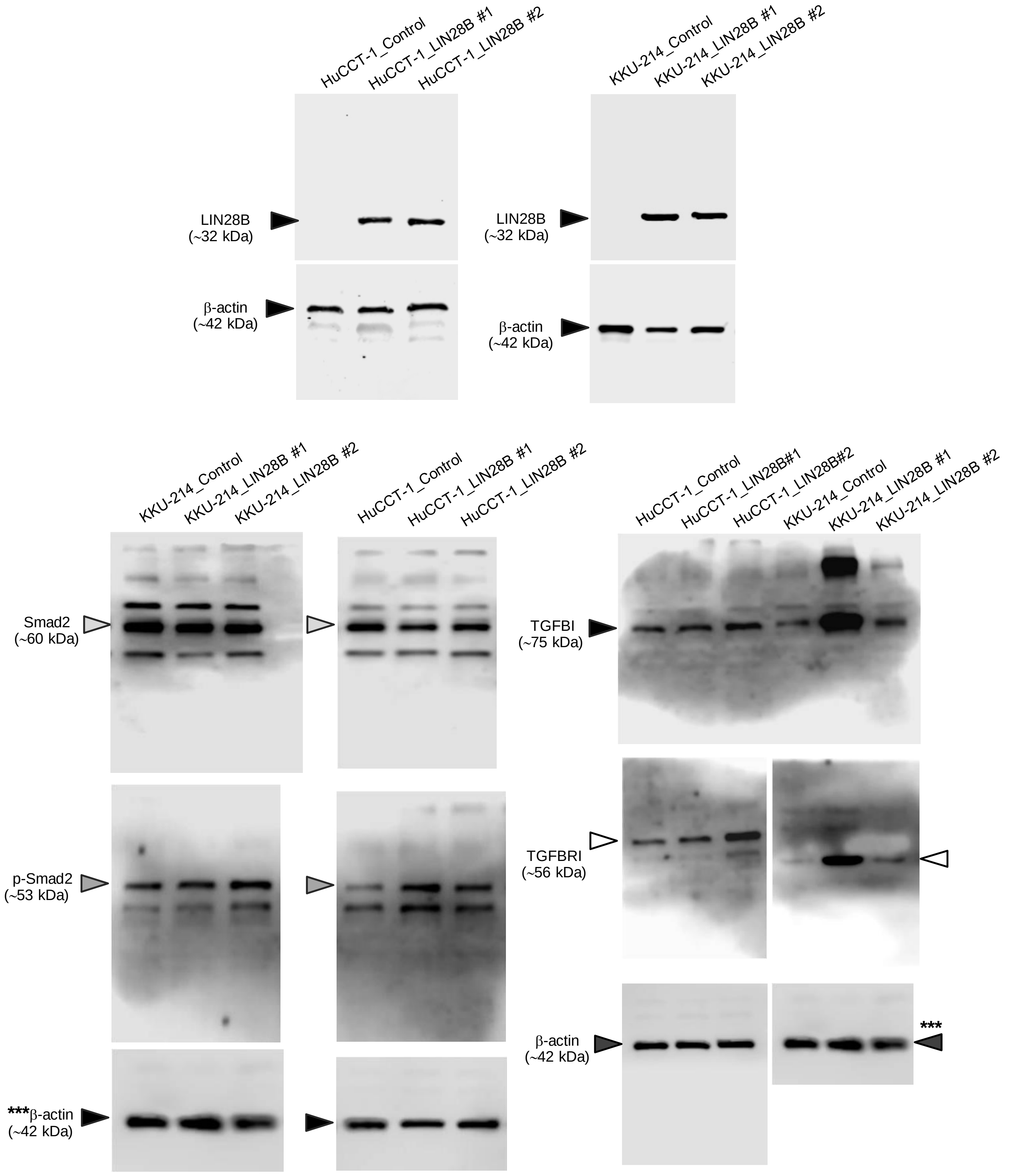

Figure 6a, western blot; TGFBI

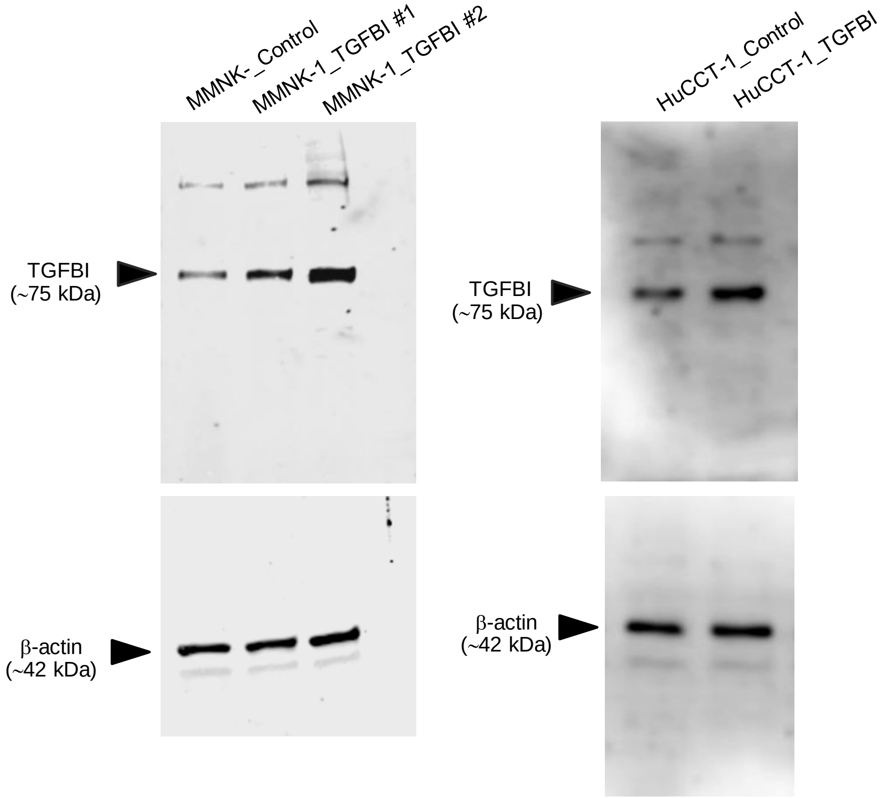

Supplementary figure 1a, western blot; LIN28B

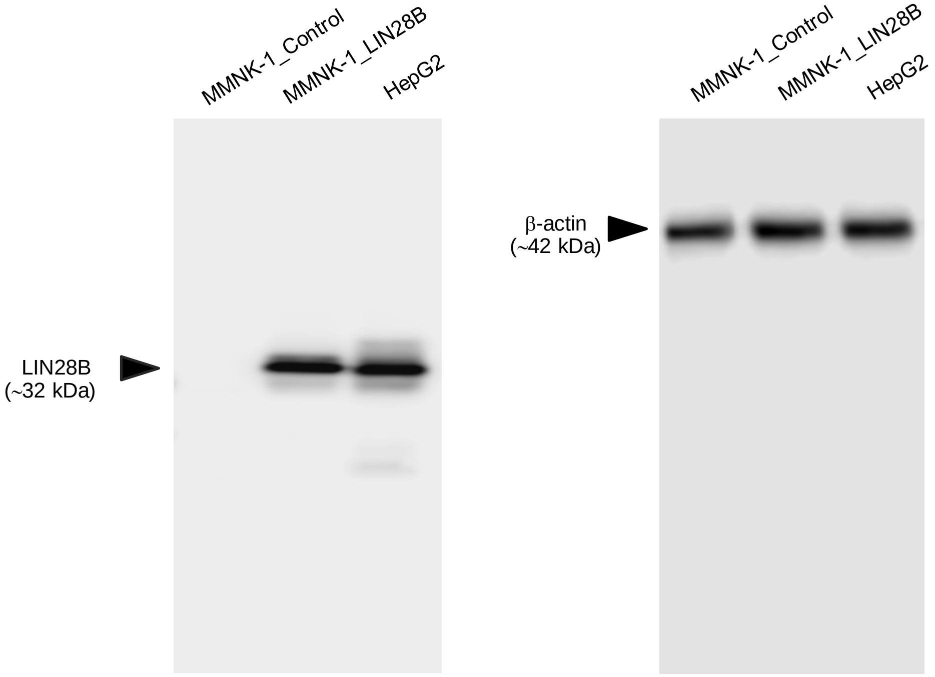

Supplementary information, western blot; Fibronectin

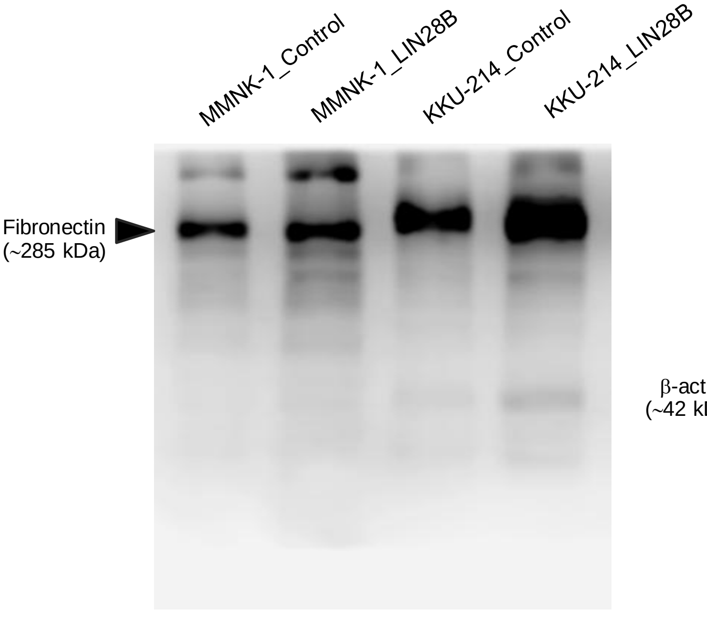

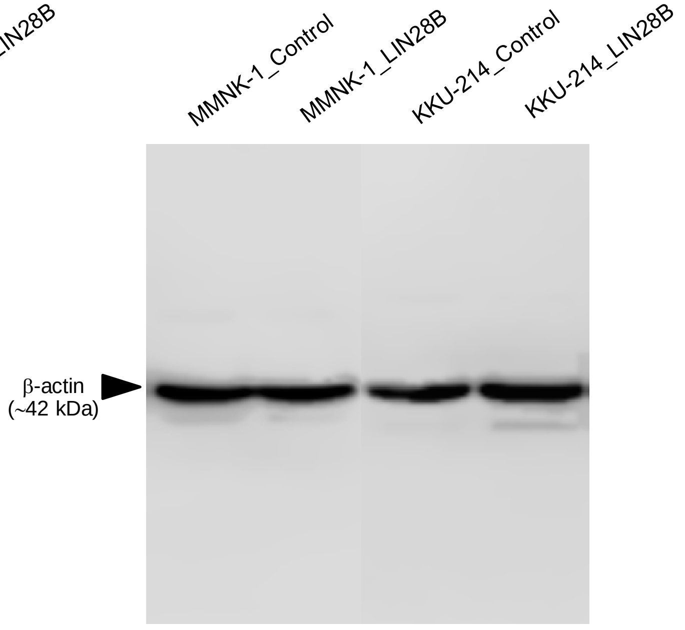

Supplement: Supplementary file 9 — Western blot [file 41417_2021_387_MOESM9_ESM.pdf]
